# Adipose Tissue and Central Nervous System Crosstalk: Roles in Pain and Cognitive Dysfunction

**DOI:** 10.3390/biomedicines14010054

**Published:** 2025-12-26

**Authors:** Juan Li, Zhixiao Li, Kun Chen, Yanqiong Wu, Xuesong Yang, Zhigang He, Hongbing Xiang

**Affiliations:** 1 Department of Anesthesiology, Hubei Key Laboratory of Geriatric Anesthesia and Perioperative Brain Health, Wuhan Clinical Research Center for Geriatric Anesthesia, Tongji Hospital, Tongji Medical College, Huazhong University of Science and Technology, Wuhan 430000, China; ethereal13@163.com (J.L.); lzx2022@tjh.tjmu.edu.cn (Z.L.); xiangfanck@163.com (K.C.); wuyanqiong-iap@taihehospital.com (Y.W.); a2579195470@163.com (X.Y.); 2 Department of Anesthesiology, Wenchang People’s Hospital, Wenchang 571300, China; 3 Key Laboratory of Anesthesiology and Resuscitation, Huazhong University of Science and Technology, Ministry of Education, Wuhan 430074, China

**Keywords:** adipose tissue, central nervous system, pain, cognitive dysfunction

## Abstract

The global obesity pandemic has unveiled adipose tissue as a pivotal, active modulator of neurological health, intricately linking metabolic dysfunction to chronic pain and cognitive decline. This review synthesizes current evidence to propose a unified “neuro-metabo-inflammatory” model of the adipose-central nervous system (CNS) axis. We articulate a framework where, in pathological states such as obesity, dysfunctional adipose tissue releases a milieu of factors—including adipokines, lipids, and extracellular vesicles—that propagate peripheral and central neuroinflammation, disrupt blood–brain barrier integrity, and impair synaptic plasticity. These processes converge to drive pain sensitization and cognitive deficits. Critically, we evaluate the clinical evidence linking visceral adiposity to multisite chronic pain and accelerated cognitive impairment, while highlighting sexually dimorphic pathways. The review moves beyond cataloging findings to prioritize the most robust mechanisms, assess evidence quality, and identify key translational gaps. We conclude by discussing emerging therapeutic strategies targeting this axis and proposing precise directions for future research to disentangle the complex temporal and spatial dynamics of adipose–CNS communication.

## 1. Introduction

The global obesity pandemic has unveiled adipose tissue as a pivotal modulator of neurological health, linking metabolic dysfunction to chronic pain and cognitive decline [1]. Adipose tissue functions as an active endocrine organ, secreting adipokines, lipids, and extracellular vesicles (EVs) that exert systemic effects [2,3]. Immunofluorescence studies have observed catecholaminergic innervation in white adipose tissue (WAT) and brown adipose tissue (BAT), providing indirect evidence of sympathetic innervation in WAT vasculature. Subsequent electron microscopy confirmed direct neural innervation of WAT adipocytes [4,5]. Adipose tissue is densely innervated by sympathetic and sensory nerves, forming a bidirectional communication circuit with the CNS. The CNS regulates adipose metabolism via sympathetic outflow, while adipose-derived signals inform the brain of peripheral energy status and inflammation levels [6,7]. Immune and endothelial cells within adipose tissue communicate with local nerve fibers, while paracrine factors from adipocyte-neuron crosstalk regulate neurotransmitter release, hemodynamics, adipocyte differentiation, and energy expenditure [8]. These findings hold significant clinical implications, offering potential therapeutic targets for metabolic and neurological disorders.

Pain, a complex condition affecting multiple systems, involves interactions between adipocytes and immune cells in adipose tissue, which modulate local and systemic immune responses to influence pain initiation and progression [9]. Epidemiological studies consistently associate visceral fat with elevated chronic pain risk and accelerated cognitive impairment [10,11]. This review advances a unified “neuro-metabo-inflammatory” model of the adipose–CNS axis, positing that dysfunctional adipose tissue is a common pathological hub for pain sensitization and cognitive decline via neuroimmune and metabolic crosstalk, highlighting urgent therapeutic opportunities.

## 2. Anatomical and Neural Foundations of Adipose–CNS Communication

### 2.1. Sympathetic Nervous Regulation of Adipose Tissue

Immunohistochemical analyses have identified tyrosine hydroxylase (TH), a marker of sympathetic neurons, in adipose tissue, revealing that sympathetic nerves are closely associated with vasculature, resembling afferent pathways [12,13]. Fasting-induced lipolysis in WAT requires sympathetic innervation and norepinephrine (NE) release from sympathetic fibers, independent of adrenal epinephrine [14], highlighting the necessity of sympathetic drive for FFA mobilization and utilization as fuel. Buettner et al. demonstrated that central insulin administration inhibits peripheral adipocyte lipolysis, indicating insulin’s dual role in adipocytes and hypothalamic suppression of sympathetic activity [15]. Collectively, these findings underscore that NE and insulin-mediated sympathetic neuron modulation govern adipocyte lipolysis. Similarly, sympathetic innervation of BAT is critical for thermogenesis, as evidenced by denervation experiments in rodents and pharmacological studies using β3-adrenergic agonists/antagonists [16,17]. Furthermore, a recent study demonstrated that applying electrical stimulation to the dorsal surface of interscapular BAT induced significant tissue temperature elevation without affecting rectal temperature. On day 2 post-sympathetic denervation surgery, electrical stimulation failed to elevate BAT temperature. This phenomenon is unlikely attributable to loss of thermogenic capacity, as neither UCP1 content nor norepinephrine-induced thermogenesis showed reduction 2 days after denervation. Pharmacological experiments revealed that BAT thermogenesis following electrical stimulation was mediated through β-adrenergic receptors. This study indicates that electrical stimulation of the BAT dorsal surface activates its thermogenic activity via sympathetic nerve-released norepinephrine [18]. The necessity of the sympathetic nervous system (SNS) for BAT functionality has been validated through both surgical and chemical denervation approaches. Thus, sympathetic innervation of adipose tissue is crucial for homeostatic control of systemic metabolism [19]. Sympathetic nerve fibers are also present within adipose parenchyma, particularly under cold exposure, which triggers sympathetic outflow and adipose tissue activation. Increased parenchymal sympathetic innervation in both WAT and BAT is essential for cold-induced upregulation of thermogenic gene expression in adipose tissues [20]. Consequently, selective sympathetic blockade in WAT and BAT disrupts cold-induced thermogenesis [21,22]. A recent report also confirmed the importance of adipose sympathetic drive for thermogenesis and systemic metabolism, demonstrating that localized sympathectomy impaired adipose thermogenesis and made mice prone to obesity [19].

Previous studies have demonstrated through chemogenetic and denervation techniques that WAT and BAT are innervated by sympathetic nerves, which are critical for tissue function and metabolic regulation [23]. However, adipose tissues exhibit cellular and functional heterogeneity across anatomical depots, categorized into white, brown, and beige adipose subtypes [24]. To delineate potential differences in sympathetic nervous system (SNS) innervation among anatomically distinct adipose depots, the Bartness group pioneered bidirectional mapping of postganglionic sympathetic innervation in WAT by injecting a retrograde tracer (FluoroGold) into WAT to label sympathetic ganglia and an anterograde tracer (DiI) into ganglia to trace sympathetic fibers surrounding adipocytes [25]. Viral transsynaptic tract-tracing methodologies have further enabled multispecies identification of central neural origins projecting to WAT [26]. Pseudorabies virus (PRV), a retrograde viral tracer, has been employed to characterize CNS innervation circuits targeting peripheral organs. Using this technique, the Bartness team revealed that dozens of neuraxis-spanning CNS loci constitute the CNS-SNS-WAT/BAT neural circuitry, providing evidence for sensory innervation of adipose tissues. Their findings demonstrated largely overlapping CNS infection regions across adipose depots, with differential infection intensities in hypothalamic nuclei, particularly highlighting the hypothalamus’s pivotal role in WAT/BAT neuroregulation [27]. Although neuronal hubs within these central circuits are broadly conserved, variations in infection density suggest depot-specific sympathetic circuit organization [26]. Notably, the suprachiasmatic nucleus, dorsomedial hypothalamic nucleus, arcuate nucleus, C1 adrenergic cells, and nucleus of the solitary tract exhibited stronger labeling in epididymal WAT (EWAT) circuits compared to inguinal WAT (IWAT) [28,29]. Conversely, IWAT circuits showed greater labeling in lateral and gigantocellular reticular nuclei. Current research supports the central melanocortin system’s critical involvement in peripheral lipid metabolism and the preoptic area’s central role in BAT thermogenesis-mediated thermoregulation [30,31].

In conclusion, sympathetic nerves densely innervate adipose tissue, releasing norepinephrine to drive lipolysis in WAT and thermogenesis in BAT. This efferent control is orchestrated by hypothalamic nuclei, forming a central sympathetic-adipose circuit. Neural tracing reveals shared command centers but depot-specific wiring patterns, indicating tailored regulatory mechanisms for different fat depots. Sympathetic input is essential for cold-induced thermogenic gene expression and metabolic homeostasis.

### 2.2. Sympathetic-Derived Signaling Factors in Adipose Tissue

Under sympathetic nervous system (SNS) activation, efferent sympathetic fibers mediate adipose metabolic homeostasis through multimodal regulatory networks: norepinephrine (NE) drives lipolytic activation, neuropeptide Y (NPY) orchestrates energy homeostasis, and ATP fine-tunes adipose microenvironmental equilibrium via purinergic receptor signaling axes. These sympathetic-derived factors reside in nerve fibers and are released upon action potential generation. Notably, NE and ATP exhibit co-transmission and coordinated action at vascular neuroeffector junctions [32]. Central SNS stimulation triggers NE release from sympathetic terminals in adipose tissue, where it binds adrenergic receptors to regulate diverse metabolic processes, including lipolysis, thermogenesis, and adipose remodeling [33,34]. Adrenergic receptor activation engages the canonical cAMP signaling pathway, mobilizing triglyceride (TAG) hydrolysis in lipid droplets. This pathway also induces WAT “browning” via beige adipocyte recruitment and BAT thermogenic gene programs, upregulating mitochondrial and oxidative genes such as uncoupling protein 1 (UCP1) [35]. β-Adrenergic receptors (β1, β2, and β3) are indispensable mediators of NE action in adipose tissue, as mice lacking these receptor subtypes exhibit metabolic dysregulation and obesity susceptibility [36,37]. During cold adaptation, BAT blood flow increases, with localized vasodilatory factors like nitric oxide (NO) proposed to enhance BAT perfusion during thermogenesis [38]. Sympathetic stimulation appears integral to this process, as NE potentiates local blood flow while upregulating nitric oxide synthase (NOS) in BAT [39,40]. Additionally, NE activates the ERK1/2-MAPK signaling cascade to initiate adipogenic transcriptional networks [41]. Subcellular localization analysis reveals that NPY co-localizes with NE in dense-core vesicles of sympathetic nerve terminals, providing a structural basis for their synergistic regulation of adipose metabolism [42].

NPY biosynthesis exhibits dual localization characteristics: while predominantly synthesized and processed within CNS neurons, peripheral sympathetic axons also demonstrate autonomous NPY production capacity, enabling direct modulation of adipocyte biological responses [43]. Mechanistically, NPY mediates signaling through selective binding to Y1/Y2 receptor subtypes, downregulating cAMP-PKA signaling via adenylyl cyclase inhibition, thereby functionally antagonizing adrenergic system activity [44]. NPY receptor activation not only suppresses adipocyte lipolysis [45] but also initiates pro-adipogenic transcriptional networks [46]. Similar to NE, NPY is stored in cytoplasmic granules at sympathetic nerve terminals. Signaling through NPY receptors promotes adipose angiogenesis and adipocyte differentiation. Furthermore, elevated circulating NPY levels and augmented receptor expression in WAT of obese mice implicate this neuropeptide in obesity-associated adipose tissue expansion. This hypothesis is further substantiated by adipose-targeted NPY receptor knockout models and pharmacological inhibition of Y2 receptors [47]. Analogous to NE and NPY, ATP functions as a sympathetic co-transmitter released from nerve terminals [48]. Upon neuronal release, ATP exerts biological effects through activation of P2X receptor family members (P2XRs) in the purinergic signaling system. Research indicates this purinergic axis profoundly influences adipose pathophysiology via multi-target regulatory networks: metabolically, it bidirectionally modulates lipogenesis and lipolysis through AMPK/mTOR pathways; energetically, it activates UCP1-mediated thermogenic programs; and immunologically, it regulates NLRP3 inflammasome activation in adipose-resident macrophages and maintains endocrine balance of adipokines (e.g., adiponectin/leptin) [49].

In conclusion, sympathetic activation releases co-transmitters NE, NPY, and ATP from efferent fibers. NE drives lipolysis and thermogenesis via β-adrenergic receptors, while NPY antagonizes adrenergic signaling and promotes adipogenesis. ATP fine-tunes the adipose microenvironment via purinergic receptors, influencing metabolism and inflammation. These factors are co-stored and act synergistically to regulate adipose tissue homeostasis.

### 2.3. Sensory Innervation in Adipose Tissue Function

Early studies identified sympathetic innervation in adipose tissue, but recent evidence highlights the presence and functional roles of sensory nerves. Fluorescent retrograde tracing with True Blue clearly visualizes sensory fibers in adipose depots. Immunohistochemical analyses further confirm sensory innervation by detecting molecular markers selectively expressed in afferent neurons, such as calcitonin gene-related peptide (CGRP) [4,50]. Subsequent meticulous studies by Bartness and colleagues demonstrated brain-projection-capable sensory fibers within adipose tissue [7,51,52]. Using herpes simplex virus-1 strain H129—an anterograde tract tracer—these studies mapped sensory pathways linking adipose depots to the brain. Functional denervation of sensory fibers in WAT revealed sensory-SNS crosstalk between inguinal WAT (IWAT) and BAT. Disruption of this sensory-SNS feedback loop altered SNS drive to specific WAT and BAT depots, suppressing sympathetic outflow and thermogenesis in BAT triggered by remote WAT lipolysis [53]. These findings establish that adipose sensory nerves detect metabolic signals within fat depots and relay them to the brain, playing a pivotal role in maintaining metabolic homeostasis.

### 2.4. Sensory-Derived Signaling Factors in Adipose Regulation

Stimulation of local sensory nerve fibers releases factors modulating adipose tissue function and systemic metabolism [54]. Calcitonin gene-related peptide (CGRP), a bioactive neuropeptide synthesized via alternative splicing of the calcitonin/CGRP gene, is produced by both central and peripheral neurons. Predominantly distributed in cardiovascular and nervous systems [55], CGRP primarily targets endothelial cells. Upon binding to calcitonin receptor-like receptor (CLR) on endothelial cells, CGRP acts as a potent vasodilator [56]. CLR signaling also promotes immune cell adhesion/migration across endothelia and regulates tissue inflammation [57]. CGRP is expressed in white adipose tissue (WAT), and its metabolic role was investigated using CGRP-knockout (CGRP^−/−^) mice. After 10 weeks of high-fat feeding, CGRP^−/−^ and wild-type (WT) mice showed comparable food intake, but CGRP^−/−^ mice exhibited reduced body weight, visceral adiposity, hepatic steatosis, and lower serum insulin/leptin levels. Enhanced glucose tolerance, insulin sensitivity, and suppressed adipocyte hypertrophy were observed in CGRP^−/−^ mice, alongside upregulated β3-adrenergic receptor, hormone-sensitive lipase, and adiponectin expression. Isoproterenol-induced glycerol release in WAT and sympathetic activity were elevated in CGRP^−/−^ mice. β-Blockade abolished the anti-obesity effects of CGRP deletion, confirming CGRP as a key regulator of systemic metabolism and energy homeostasis beyond its cardiovascular roles [58]. Thus, sensory nerve-derived CGRP critically governs adipose vascular homeostasis, inflammation, and metabolic regulation. Substance P (SP), a neuropeptide released by peripheral sensory nerves, acts as a vasodilator and immunomodulator [59]. Binding to neurokinin 1 receptor (NK1R)—expressed by adipocytes and immune cells—SP stimulates pro-inflammatory cytokine production in immune cells, implicating it in adipose inflammation during obesity and metabolic dysfunction [60]. Elevated SP and NK1R expression was observed in mesenteric adipose depots of colitis patients [61]. Mechanistically, SP promotes adipocyte lipolysis, suppresses lipid droplet accumulation during differentiation, and blocks insulin-mediated fatty acid uptake [62].

In conclusion, adipose tissue contains sensory nerves that form afferent pathways to the brain and engage in sensory-SNS crosstalk to regulate metabolism. Sensory-derived neuropeptides—CGRP and Substance P—modulate adipose function: CGRP deletion improves obesity and metabolic profiles, while Substance P promotes lipolysis and inflammation via NK1R.

This schematic illustrates the anatomical foundation of bidirectional communication between adipose tissue and CNS. Efferent sympathetic outflow from key hypothalamic and brainstem nuclei regulates WAT/BAT metabolism, controlling lipolysis and thermogenesis. Afferent sensory feedback from adipose depots projects to central sensory circuits, informing the brain of peripheral metabolic and inflammatory status. This reciprocal loop establishes a closed neural circuit for rapid physiological adaptation, which becomes dysregulated in metabolic disease.

### 2.5. Adipokines-Derived Signaling in Central Neural Regulation

Adipocyte-derived neurotrophic factors are crucial for sympathetic nervous system (SNS) innervation of adipose depots. Neurotrophin-3 (NT-3) and its receptor TrkC promote sympathetic neurite outgrowth; enhancing this axis increases innervation, beige adipogenesis, and cold-induced thermogenesis, while its impairment reduces cold tolerance and promotes obesity [63]. Similarly, neuregulin 4 (NRG4) and nerve growth factor (NGF) support sympathetic plasticity in response to cold exposure. Conversely, the adipokine leptin is essential for maintaining sympathetic nerve density in fat, regulating structural plasticity via top-down neural circuits [64]. These factors collectively govern SNS-driven energy expenditure.

Adipocyte-derived metabolites directly influence neural activity. Chronic fatty acid infusion suppresses peripheral sympathetic nerve activity. Adipose-produced endocannabinoids (ECs) inhibit sympathetic outflow and thermogenesis, potentially contributing to metabolic dysfunction. Furthermore, pro-inflammatory cytokines (e.g., TNFα, IL-1β) from adipose tissue macrophages can enhance central sympathetic outflow [65,66,67]. However, under chronic inflammatory conditions like obesity, local cytokines may instead repel sympathetic fibers or cause neuropathy [68].

Adipokines and lipids also regulate sensory afferents, shaping adipose-to-brain communication. Leptin acts locally on sensory nerves in white adipose tissue (WAT); its deletion in vagal afferents disrupts central control of feeding and promotes obesity [69]. Adipose-derived lipids, including free fatty acids and arachidonic acid derivatives, are essential for activating sensory circuits that link WAT lipolysis to brown adipose tissue thermogenesis. Other factors like vascular endothelial growth factor (VEGF) promote angiogenesis alongside the innervation of adipose tissue [70,71].

**Figure 1:**
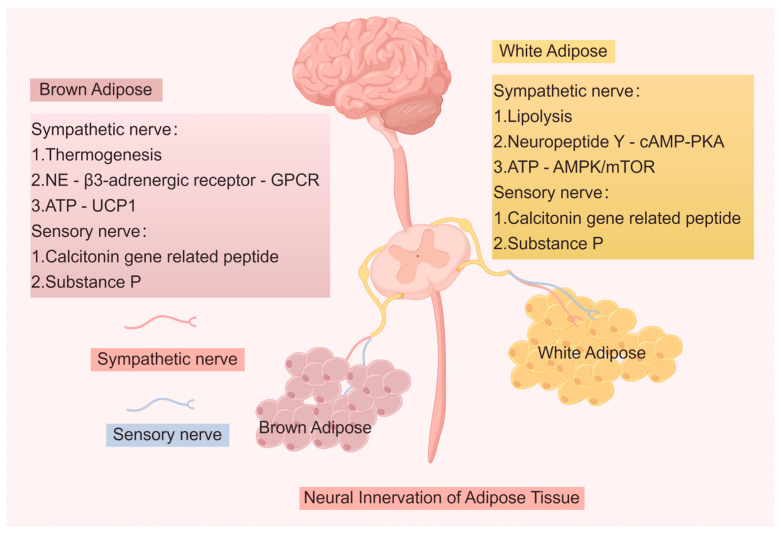
Neural Circuits of the Adipose–CNS Axis: Bidirectional Innervation and Central Regulation.

**Figure 2:**
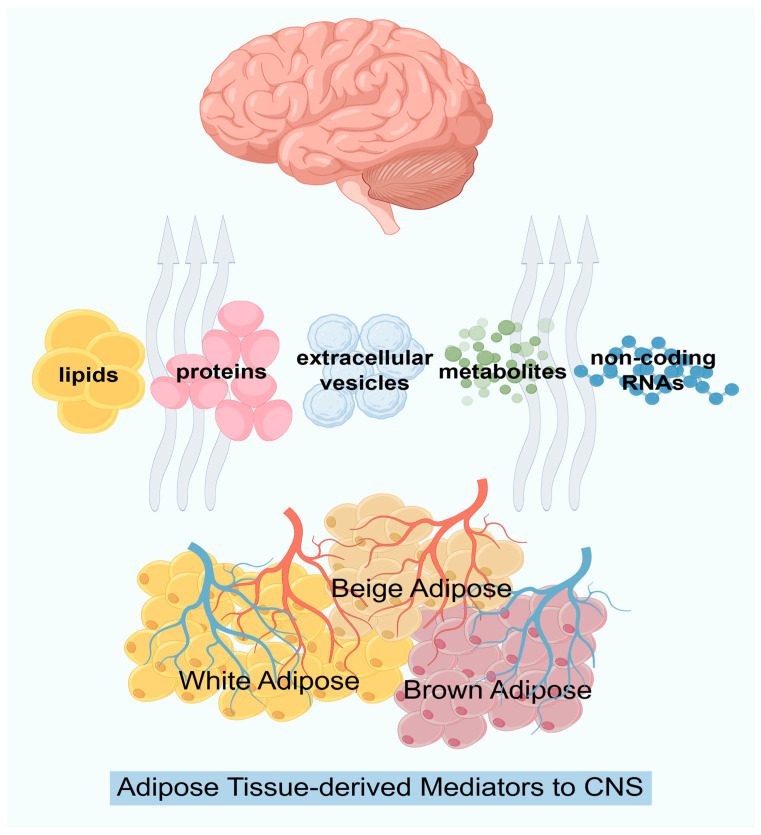
Adipose Tissue-derived Mediators to CNS.

In summary, adipose tissue is a rich source of signals that bidirectionally modulate neural circuits (Figure 1), critically integrating peripheral metabolic status with central regulatory commands (Figure 2).

This integrative diagram categorizes the key signaling molecules released by dysfunctional adipose tissue and their pathways to the CNS. Adipose tissue is a dynamic secretory organ that can secrete lipids, proteins, extracellular vesicles, metabolites and non-coding RNAs to regulate other organs of the human body and communicate with the central nervous system.

## 3. Advances in Adipose Tissue-Pain Crosstalk

**Figure 3:**
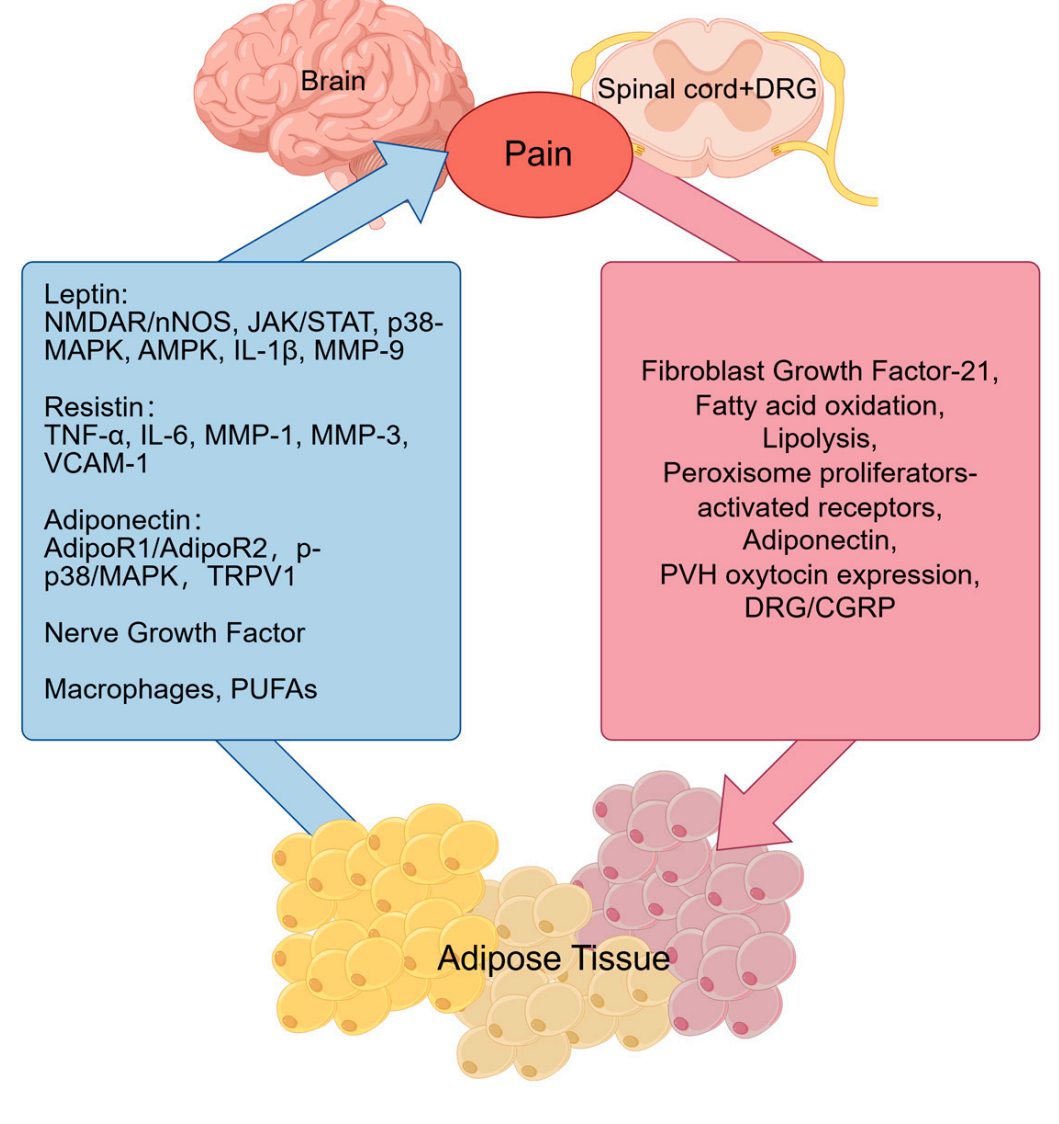
Pathophysiological Cascade: From Adipose Dysfunction to Chronic Pain.

This section delves into the mechanisms by which dysfunctional adipose tissue acts as a critical upstream driver within the neuro-metabo-inflammatory axis to initiate and perpetuate chronic pain (TABLE1, Figure 3). Adipose tissue communicates with the central nervous system (CNS) by secreting a diverse array of mediators, including adipokines, cytokines, and extracellular vesicles. These signals can directly modulate neuronal activity and shape neuroinflammatory responses, thereby regulating pain perception and transmission. Dysregulated secretion of these bioactive molecules, particularly in states like obesity, leads to a propagated inflammatory state that disrupts normal nociceptive processing. This crosstalk exemplifies the model’s core premise: metabolic disturbances in adipose tissue generate an inflammatory mediator milieu that adversely impacts neuronal function and contributes to pain pathologies.

### 3.1. Adipose Tissue Modulation of Pain

The hypothalamic–pituitary–adrenal (HPA) axis, hypothalamic-pituitary-gonadal (HPG) axis, and adipose-derived signaling are intricately linked to pain pathophysiology. The HPA axis, a core endocrine pathway regulating stress responses, is dysregulated in chronic pain conditions [72]. The HPG axis critically modulates pain perception with sex-specific effects mediated by estrogen and testosterone.

Leptin, a 16 kDa adipokine primarily synthesized in white and brown adipose tissue under leptin gene regulation, is also expressed in non-adipose tissues. Leptin receptors (LepR), distributed in the hypothalamus, dorsal root ganglia (DRG), heart, liver, and reproductive organs, exhibit dual pro- and anti-inflammatory properties [73], modulating systemic inflammatory markers and cytokine production linked to chronic pain. Emerging evidence highlights leptin’s multifaceted role in nociception [74]. Leptin influences pain through central and peripheral mechanisms. Hu et al. investigated nociceptive responses in diet-induced obese mice, revealing preserved thermal nociception but attenuated leptin-induced STAT3 phosphorylation and reduced inflammatory pain sensitivity. Three-week dietary normalization partially restored nociceptive responses alongside decreased adiposity and plasma leptin [75]. Spinal leptin signaling blockade prevents and reverses neuropathic pain behaviors in peripheral nerve injury models. Nerve injury upregulates leptin and LepR expression in spinal dorsal horn, where leptin enhances *N*-methyl-D-aspartate receptor (NMDAR) expression via JAK/STAT signaling and promotes IL-1β production. Genetic leptin deficiency and exogenous leptin administration respectively abolish and mimic injury-associated pain behaviors and cellular changes [76]. Maeda et al. demonstrated leptin’s essential role in tactile allodynia. Leptin-deficient ob/ob mice failed to develop nerve injury-induced allodynia. Injured nerves showed increased leptin transcription and phospho-STAT3+ macrophage recruitment. Leptin-treated macrophages upregulated MMP-9 and iNOS mRNA, restoring allodynia in ob/ob mice [77]. Clinically, elevated serum leptin correlates with chronic pain in arthritis [78], fibromyalgia, and migraine [79], while reduced levels associate with chronic low back pain [80]. Synovial fluid leptin elevation exacerbates shoulder pain severity, and preoperative hyperleptinemia predicts reduced pain thresholds and increased postoperative analgesic requirements [81].

Adiponectin, a pleiotropic adipokine predominantly synthesized in adipose tissue, crosses the blood–brain barrier and exerts systemic effects across multiple organs including the liver, muscle, heart, spinal cord, pituitary, and brain. These actions are mediated via specific binding to adiponectin receptors 1 (AdipoR1) and 2 (AdipoR2). Adiponectin exhibits multifaceted biological properties, including anti-inflammatory, antithrombotic, antiatherogenic, antiapoptotic, and insulin-sensitizing effects. Iannitti et al. [82] demonstrated in a rat peripheral inflammation model that intrathecal adiponectin administration exerts anti-inflammatory and antinociceptive effects at the spinal level through AdipoR1/AdipoR2. Notably, spinal adiponectin levels are reduced in obese rats with inflammatory hyperalgesia [83]. Sun et al. investigated adiponectin’s role in thermal nociception, revealing its modulation of heat pain sensitivity via activation of phosphorylated p38 mitogen-activated protein kinase (p-p38 MAPK) and transient receptor potential vanilloid 1 (TRPV1) in peripheral and central compartments—including dorsal root ganglion (DRG) neurons, spinal microglia, and somatosensory cortical neurons—following partial sciatic nerve ligation [84]. These findings establish adiponectin as a dual modulator of pain through peripheral and central mechanisms. Clinical studies report elevated serum adiponectin levels in migraine patients compared to controls [85], while increased synovial fluid adiponectin correlates with aggravated shoulder pain. These observations suggest dysregulated adiponectin signaling may contribute to pain pathophysiology, underscoring its therapeutic potential in pain management.

Resistin, an adipokine involved in lipid metabolism, is primarily secreted by adipose tissue and human monocytes. Its pro-inflammatory role has garnered increasing attention in recent years. Resistin contributes to various inflammatory disorders, including obesity-associated subclinical inflammation, atherosclerosis, cardiovascular diseases, non-alcoholic fatty liver disease (NAFLD), rheumatic conditions, malignancies, asthma, inflammatory bowel disease, and chronic kidney disease [86]. In obesity, elevated resistin levels exacerbate adipose tissue inflammation by activating macrophages and enhancing pro-inflammatory cytokine secretion, perpetuating chronic low-grade inflammation [87]. In arthritis, resistin primarily mediates pathology through its pro-inflammatory properties. Osteoarthritis patients exhibit higher synovial fluid resistin levels, positively correlated with inflammatory markers such as IL-6, MMP-1, and MMP-3. Resistin promotes synovial fibroblast activation by suppressing miR-149 and miR-381 expression, thereby upregulating IL-1β, TNF-α, and vascular cell adhesion molecule 1 (VCAM-1), which enhances monocyte adhesion to synovial fibroblasts [88,89]. Postoperative pain intensity is also influenced by serum resistin levels. Resistin directly or indirectly stimulates macrophages to release TNF-α, IL-1β, and IL-6, which sensitize peripheral nociceptive neurons, leading to persistent pain and hyperalgesia [90].

Nerve Growth Factor (NGF) and its receptors have emerged as novel therapeutic targets for neuropathic pain. Produced by both white (WAT) and brown adipose tissue (BAT), NGF modulates lipolysis and thermogenesis. As a pro-inflammatory cytokine, NGF mediates pain signaling and induces neural sensitization. NGF injection-induced hyperalgesia involves peripheral mechanisms (e.g., mast cell degranulation and serotonin release) [91] and central mechanisms (e.g., NMDA receptor and TRPV1 activation) [92]. Inflammatory cytokines such as IL-1β and TNF-α further amplify NGF-driven thermal and mechanical hypersensitivity. Notably, TNF-α treatment significantly induces NGF gene expression in human adipocytes [93], underscoring NGF as a key pro-inflammatory adipokine.

The research strives to examine the connection between the visceral adiposity index (VAI) and persistent pain in adult participants of the 1999–2004 NHANES and to assess VAI as a novel indicator for risk assessment and preliminary diagnosis in individuals with chronic pain. This study identified a significant association between VAI and chronic pain. Higher VAI levels were associated with increased prevalence of chronic pain, and there was a nonlinear relationship between the two. VAI was superior to BMI in predicting chronic pain and could be used as a potential predictor of chronic pain risk [94]. A large prospective, population-based cohort study quantified visceral adipose tissue and subcutaneous adipose tissue by using abdominal MRI scans. The research identified that abdominal adipose tissue was associated with chronic musculoskeletal pain, suggesting that excessive and ectopic fat depositions may be involved in the pathogenesis of multisite and widespread chronic musculoskeletal pain. The identified stronger effects in women than men may reflect sex differences in fat distribution and hormones [95].

### 3.2. Immune and Lipid Mediators in Adipose-Pain Crosstalk

Adipose-resident macrophages, particularly within the infrapatellar fat pad (IFP), are implicated in obesity-driven osteoarthritis (OA) progression. IFP macrophages exhibit a mixed pro-/anti-inflammatory phenotype, potentially influencing knee pathology through secretion of inflammatory mediators and modulation of IFP fibrosis [96]. M1 macrophages exacerbate arthritis by promoting inflammation and pain sensitization, whereas M2 macrophages exert anti-inflammatory effects. Therapeutic strategies targeting macrophage polarization—notably via adipose-derived mesenchymal stem cells (AD-MSCs)—may ameliorate arthritic pathology [97]. In neuropathic pain, dysregulated neuroimmune interactions involve macrophage-derived inflammatory cytokines and chemokines that activate peripheral cells and recruit leukocytes, perpetuating pain chronification [98]. Hypertrophic scars often cause localized pain. Research has found that fat grafting can alleviate the localized pain. Within the transplanted adipose tissue, there exists a cell population characterized as CD3^−^CD4^−^CD304^+^, whose abundance is highly correlated with short-term postoperative pain improvement. These cells are characterized by the absence of the hematopoietic marker CD45, whereas they express CD90 and CD34, which characterize mesenchymal stem cells (MSCs); the concomitant presence of CD10 and CD73 in the plasma membrane supports a function of these cells in pain reduction. Thus, Enrichment of specific adipose mesenchymal subpopulations enhances the therapeutic efficacy of fat grafting in alleviating localized pain syndromes [99]. In the other study, adipose-derived stem cell (ADSC) exosomes inhibited expression of fibrosis-related molecules such as α-smooth muscle actin, collagen I (COL1) and COL3 and inhibited the transdifferentiation of myofibroblasts. MicroRNA-125b-5p (miR-125b-5p) is highly expressed in ADSC exosomes and binds to the 3′ untranslated region of Smad2, thus inhibiting its expression, speeding up wound healing and preventing scar formation [100]. Autologous adipose tissue grafting is a promising and safe modality for the treatment of the painful scar. There is an abundance of low-level evidence to support its use as an alternative treatment, but there is a lack of high-level evidence at present to support its standard use [101]. Therefore, more clinical and basic research is needed to support the application of fat transplantation to relieve localized pain.

Polyunsaturated fatty acid (PUFAs), essential fatty acids classified into ω-3 and ω-6 families, are critical for membrane integrity and function, with metabolites actively regulating inflammatory and nociceptive processes. ω-3 PUFAs (e.g., EPA, DHA) alleviate rheumatoid arthritis pain and morning stiffness by suppressing pro-inflammatory cytokine production. Their anti-inflammatory and pro-resolving effects are mediated via specialized pro-resolving mediators (SPMs) such as resolvins, which restore tissue homeostasis [102]. In diabetic neuropathy, ω-3 PUFAs improve symptoms by upregulating antioxidant pathways and mitigating oxidative stress [103]. Conversely, excessive ω-6 PUFA intake exacerbates inflammation and pain; diets high in ω-6 PUFAs induce sustained hyperalgesia and peripheral nerve injury in murine models [104]. Clinically, optimizing the ω-6/ω-3 ratio may represent a strategic intervention for pain management.

### 3.3. Impact of Pain on Adipose Tissue

Spinal cord injury (SCI) induces pathological adipose tissue expansion (often leading to obesity) and ectopic lipid accumulation in organs critical for glucose and insulin metabolism, such as the liver, heart, and skeletal muscle [105]. Post-SCI ectopic lipid deposition is also observed in these organs. Adipose tissue maintains energy homeostasis by mobilizing triglycerides into glycerol and free fatty acids via lipolysis, which serve as fuel for brown adipose tissue (BAT), heart, liver, and muscle. Disrupted energy balance—when adipose tissue cannot expand metabolically to accommodate excess lipid storage—triggers ectopic lipid accumulation in insulin-sensitive organs. Recent studies highlight the pathophysiology of post-SCI adipose dysfunction. SCI reduces serum fibroblast growth factor-21 (FGF-21) levels in mice and impairs FGF-21 signaling in the liver and adipose tissue. FGF-21 activates FGFR1/β-klotho receptors to stimulate adiponectin release from adipose tissue, which subsequently acts on the liver and skeletal muscle. In both normal and high-fat diet (HFD)-fed mice with spinal transection, the FGF-21/adiponectin axis is disrupted, characterized by suppressed FGF-21 receptor expression and reduced adiponectin levels in adipose tissue and serum. This impairment likely contributes to post-SCI metabolic dysregulation [106]. SCI also promotes epididymal white adipose tissue (eWAT) lipolysis via sensory nerve activation, elevating serum glycerol and non-esterified fatty acid levels through adipose mobilization [107]. Furthermore, peripheral neuropathy alters adipose function in a sex-specific manner. Female mice with sciatic nerve injury exhibit modified lipolysis, enhanced fatty acid oxidation, increased systemic energy expenditure, and altered adipokine secretion, collectively impacting glucose/insulin metabolism. In contrast, males show reduced energy expenditure, diminished unsaturated fatty acid levels, and upregulated secretion of regenerative/oxidative stress-related factors (e.g., peroxisome proliferator-activated receptors and adiponectin) in adipose tissue [108]. These findings underscore adipose tissue’s pivotal role in interorgan communication and metabolic adaptation following neural injury.

### 3.4. Neural Circuits and Sex-Specific Adipokine Dynamics in Pain-Adipose Crosstalk

Adipose tissue receives dual innervation from sympathetic and sensory nerves, with spinal and supraspinal sympathetic-sensory feedback loops modulating lipolysis. White adipose tissue (WAT) depends on sympathetic nervous system (SNS) activation for lipid mobilization. In neuropathic pain models, upregulated adrenergic receptor expression in WAT correlates with altered metabolism, energy expenditure, and secretory function, driving lipid/energy dysregulation [7]. A study by Tedeschi et al. demonstrated that spinal cord injury (SCI) at T12 exacerbates epididymal WAT (eWAT) lipolysis in mice, elevating serum glycerol and non-esterified fatty acids via adipose mobilization, despite preserved sympathetic tone. Concurrently, increased α2δ1 subunit expression in calcitonin gene-related peptide (CGRP)-positive L3-5 dorsal root ganglia (DRG) suggests sensory neuropathy-induced lipolytic dysregulation, implicating modified sympathetic-sensory feedback [7]. While adipose functional changes (e.g., enhanced lipolysis, altered adipokine secretion) are observed in pain models, mechanistic validation remains limited, necessitating further investigation.

The hypothalamic paraventricular nucleus (PVH) integrates feeding behavior and energy balance through neuropeptides such as neuropeptide Y (NPY), α-melanocyte-stimulating hormone (α-MSH), and oxytocin. Pseudorabies virus tracing reveals PVH-to-adipose sympathetic projections mediating WAT lipid mobilization. As a hub in long-loop sympathetic-sensory feedback circuits connecting WAT/BAT to the brain, PVH critically regulates adipose neuroexcitability. PVH also interfaces with ascending pain transmission and descending analgesic pathways. Prefrontal cortical oxytocinergic projections modulate nociception and anxiety-related behaviors [107,108]. Chronic inflammatory and neuropathic pain upregulates PVH oxytocin expression, selectively activating spinal dorsal horn glutamatergic neurons. Emerging evidence suggests parallel PVH pathways may link pain modulation to sympathetic adipose activation, with oxytocinergic neurons potentially mediating dual roles in analgesia and lipolysis—mechanisms requiring further exploration [109].

Adipose leptin and adiponectin secretion exhibit sex-dimorphic patterns following peripheral nerve injury. In female mice with chronic constriction injury (CCI) of the sciatic nerve, leptin expression is upregulated [110]. Conversely, CCI induces reactive increases in leptin and its receptor within rat DRG neurons. Exogenous leptin alleviates pain and suppresses DRG IL-6/TNFα expression. Strikingly, adiponectin upregulation post-injury occurs exclusively in males, paralleling peroxisome proliferator-activated receptor gamma (PPARγ) induction. Low adiponectin levels correlate with diabetic neuropathy [111], while adiponectin deficiency exacerbates thermal hypersensitivity [84]. Reduced mechanical pain sensitivity in males may reflect adiponectin’s protective effects. SCI reduces leptin and adiponectin mRNA expression, with disrupted hepatic FGF21-adiponectin signaling and molecular adaptations potentially explaining adiponectin resistance despite transcriptional upregulation. Reduced adipose mass in SCI mice—consistent with leptin’s positive correlation to adiposity—may partially account for diminished leptin levels [112]. Sex hormones regulate adipose tissue metabolism in a depot- and sex-specific manner by activating receptors that modulate lipolysis and lipogenesis. Levels of 17beta-estradiol (17beta-E2) were higher in inguinal than in other fat depots, with no significant sex difference in tissue concentration. The expression of androgen receptor (AR), estrogen receptor alpha/beta (ER-α/β) depended more on anatomical location than sex, being generally higher in visceral than subcutaneous depots. ER-α predominated over ER-β, while progesterone receptor expression was consistent across depots and sexes. In 3T3-L1 adipocytes, 17beta-E2 increased ER-α and AR expression, indicating estrogen’s role in modulating local hormonal signaling [113]. Adipose tissue also exhibits sex-specific responses to nerve injury. Females show enhanced lipolysis, fatty acid oxidation, energy expenditure, and steroid secretion affecting glucose metabolism. In contrast, males exhibit increased glycolysis, reduced energy expenditure, and lower unsaturated fatty acids. Male adipose tissue promotes regenerative molecules, oxidative stress defense, and activates PPAR-γ and adiponectin [106]. Pronounced sexual dimorphism exists in visceral adipose tissue (VAT) T cells. Male VAT contains more T cells, which differ from female VAT T cells in phenotype, transcriptome, and chromatin accessibility. Increased inflammation in male VAT recruits T cells via the CCL2-CCR2 axis. Androgen regulates a unique IL-33-producing stromal cell population in male VAT, paralleling local T cell expansion. Sex hormones shape the transcriptional landscape of VAT T cells in a BLIMP1-dependent manner, with the sex-specific tissue niche ultimately limiting adipose inflammation [114].

This figure presents a stepwise model of how adipose tissue dysfunction promotes chronic pain. The cascade begins with adipose expansion and inflammation, particularly in visceral depots, leading to the release of pro-nociceptive mediators. These signals cause peripheral sensitization by activating nociceptors and local macrophages, and central sensitization via blood-spinal cord barrier disruption and microglial activation in the spinal cord. This results in neural circuit dysregulation in pain-processing brain regions, culminating in the clinical phenotype of chronic pain.

## 4. Adipose Tissue Regulates Cognitive Dysfunction

**Figure 4:**
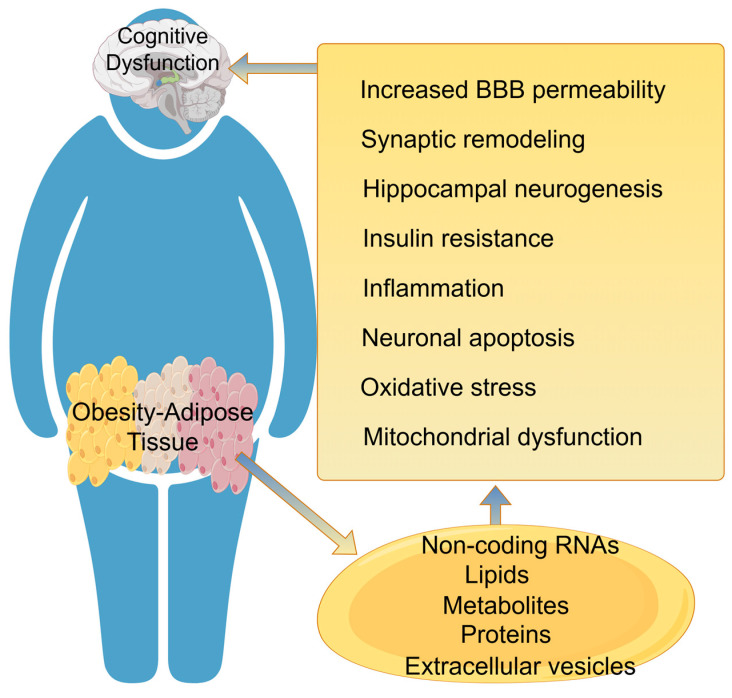
Pathophysiological Cascade: From Adipose Dysfunction to Cognitive Decline.

This section explores how adipose tissue dysfunction propagates through the neuro-metabo-inflammatory axis to impair cognitive function (Table 1, Figure 4), sharing mechanistic parallels with pain pathways. As an endocrine organ, adipose tissue secretes factors like leptin and adiponectin, which are involved in maintaining brain cognitive and memory function. However, in dysfunction, it also releases inflammatory cytokines that can cross the blood–brain barrier, activate central immune cells, and induce chronic neuroinflammation, particularly damaging hippocampal neurons. Notably, research has established a causal relationship between visceral fat and Alzheimer’s disease risk, positioning adipose tissue as a powerful upstream modifier of brain health. This shared inflammatory mechanism, originating from adipose tissue and converging on CNS vulnerability, strongly reinforces the integrative model linking metabolic dysregulation to neurological outcomes.

### 4.1. Changes in the Structure and Function of the Blood–Brain Barrier

The blood–brain barrier (BBB) is a barrier phenotype obtained by the interaction of brain endothelial cells with surrounding cells and the release of soluble factors from astrocytes. This barrier restricts uncontrolled diffusion of large molecules between blood and the brain. The action of adipose tissue-derived cytokines or adipokines increases the permeability of the BBB [115], causing structural and functional changes in the central nervous system and promoting inflammation. Leptin, resistin, and adiponectin, among other pro-inflammatory adipokines, activate central immune cells by crossing the BBB, leading to a decline in cognitive function [116]. In obese patients, obesity-related inflammation depends on the NF-κB pathway, increases pro-inflammatory protein expression, and disrupts BBB integrity by decreasing tight junction protein expression [117]. Studies have found that feeding young male SD rats a high-fat diet for 90 consecutive days increases the permeability of the BBB to low molecular weight exogenous tracer fluorescein sodium in the hippocampus, which is associated with low expression of tight junction proteins claudin-5, claudin-12, and occludin mRNA. This study also links the increased permeability of the BBB with hippocampus-dependent learning and memory impairment [118]. In addition, compared to ischemic mice fed a standard diet, 10-week-old male C57 BL/6 mice fed a cholesterol-rich diet showed increased extravasation of immunoglobulin (IgG) in the frontal cortex, enhancing the effect of ischemia on BBB permeability. Aging obesity exacerbates cognitive decline, especially in the hippocampus. Another study fed young and old male C57 BL/6 mice a standard or high-calorie diet, and found that cognitive impairment in obese and elderly mice is associated with reduced microvascular density and pericyte coverage in the hippocampus and cerebral cortex [117].

### 4.2. Hippocampal Neurogenesis

The hippocampus plays an important role in cognitive processes such as learning and memory, and the adipogenic fatty acid factor is involved in regulating hippocampal neurogenesis. Evidence from animal models has shown that compared with wild-type mice, the proliferation and differentiation of neural stem cells in the dentate gyrus of adiponectin-deficient mice are inhibited, and the number of newly generated granule neurons is reduced, while supplementing adiponectin (0.5 μg/μL) can effectively promote neurogenesis. Adiponectin promotes hippocampal neurogenesis by activating the p38 mitogen-activated protein kinase (MAPK)/GSK-3β/β-catenin signaling pathway, which enhances the proliferation of neural stem cells [119,120]. Results from adiponectin receptor (AdipoRon) intervention in male mice showed that low-dose AdipoRon intervention (20 mg/kg) can increase the level of serum brain-derived neurotrophic factor, promote hippocampal neuron proliferation, and improve cognitive function. However, high-dose AdipoRon (50 mg/kg) inhibits hippocampal neuron proliferation, differentiation, and survival, thereby impairing cognitive functions such as spatial recognition in mice [121]. Therefore, appropriate doses of adiponectin can improve cognitive function by protecting hippocampal neurogenesis.

Leptin can also regulate hippocampal neurogenesis. Evidence from animal models has shown that supplementation with leptin (1 mg/kg) can promote adult and aged mouse hippocampal dentate gyrus neurogenesis by activating the PI3K/AKT and JAK/STAT3 signaling pathways, promoting the proliferation and differentiation of new neurons [122,123]. Studies have found that high-fat diet mice have suppressed hippocampal neurogenesis, and their social cognitive and passive learning abilities significantly decreased. Leptin intervention (10~100 ng/mL) can reverse hippocampal neurogenesis inhibition and improve related cognitive functions by activating the STAT3/AMPK/ERK signaling pathway [122,123]. It is noteworthy that there are gender and age differences in the reduction in cognitive-related hippocampal neurogenesis caused by a high-fat diet, that is, high-fat diet has no effect on neurogenesis in male and adult animals, only in females and adolescents [124,125]. It is currently recognized that maternal obesity, whether before or during pregnancy, will have a negative impact on fetal brain development and increase the risk of cognitive and neurobehavioral disorders in offspring. In infancy and adolescence, obesity remains a limiting factor for healthy neurodevelopment, particularly affecting executive functions, while attention, visual–spatial abilities, and motor skills are also affected. In middle age, obesity appears to accelerate brain aging, which may increase the risk of developing neurodegenerative diseases in old age, such as Alzheimer’s disease.

### 4.3. Synaptic Plasticity

Synaptic plasticity is the neurostructural basis for advanced cognitive function, and its impairment is manifested structurally as damage to synaptic structure and functionally as abnormal long-term potentiation (LTP), resulting from functional disorders in NMDAR, AMPAR, and potassium ion channels. Leptin can effectively enhance LTP after high-frequency stimulation in the dentate gyrus of rodents and inhibit long-term depression induced by low-frequency stimulation [126]. Additionally, leptin deficiency leads to a significant reduction in complexity, such as dendritic length and spine density, in cerebellar granule neurons of mice, as well as a decrease in the basal synaptic transmission of the Schaefer collateral pathway in the hippocampus and an increase in presynaptic release probability. The levels of the GluA1 subunit of the glutamate receptor, as well as the GluN1 and GluN2A subunits, are reduced, and LTP is impaired. At the same time, the lack of leptin leads to the inhibition of the activity of the AdipoR1/AMPK/GSK-3β/cAMP response element-binding protein signaling pathway, and spatial learning and recognition memory abilities are significantly reduced [127,128]. Leptin is also associated with hippocampal synaptic plasticity. Correlative data have shown that leptin can regulate synaptic plasticity in the hippocampal CA1 region. Animals lacking or insensitive to leptin exhibit hippocampal synaptic plasticity and cognitive impairment, while leptin supplementation can enhance NMDAR function, increase hippocampal LTP, and improve memory function [128]. Research has shown that leptin can enhance NMDAR-mediated Ca2+ influx by activating MAPK and Src tyrosine kinases, increasing synaptic plasticity, and the disruption of this process is related to cognitive impairment associated with obesity [128]. Interestingly, feeding obese rodents a high-calorie diet inhibits the transport of leptin and other neurofat molecules across the blood–brain barrier, molecules that can promote synaptic plasticity and cognitive function. This functional impairment is accompanied by the activation of signal transduction and transcription activation factors (Signal Transducer and Activator of Transcription 3, STAT-3), which is one of the main signaling pathways controlled by the full-function leptin receptor subtype ObRb [129].

Inflammatory factors in adipose tissue also participate in regulating hippocampal synaptic plasticity. Exposure to high levels of IL-1β can lead to significant inhibition of learning function and synaptic plasticity, while adipose tissue transplantation can promote cognitive and synaptic changes. IL-1β levels are associated with obesity and cognitive impairment, and inhibiting hippocampal IL-1 receptors can prevent synaptic and cognitive dysfunction [130]. TNF-α has a dose-dependent regulatory effect on synaptic development. Exposure of hippocampal CA1 slices to low concentrations of TNF-α can promote activity-dependent and homeostatic plasticity by promoting AMPA receptor embedding. Inhibition of TNF-α before amyloidosis can prevent synaptic defects in an AD model [131].

### 4.4. Neuroinflammation

The increase in adipocytes triggers adipocyte differentiation, a process known as adipogenesis, followed by the infiltration of immune cells (including macrophages, neutrophils, and T lymphocytes) in adipose tissue. These processes have been identified as the main sources of cytokines and adipokines, which are major contributors to obesity-related systemic inflammation [132]. The systemic inflammation originating from adipose tissue significantly alters the levels of adipokines, including leptin, resistin, and adiponectin. This process produces pro-inflammatory mediators or adipokines (leptin, adiponectin, resistin, TNF-α, IL-1β, -6 and -8, insulin-like growth factor 1, MCP-1, and visfatin) [133]. High-fat diets have been shown to induce neuroinflammation by activating microglia and astrocytes and increasing pro-inflammatory cytokines/mediators in the hippocampus of mice, such as cyclooxygenase-2, TNF-α, IL-1-β, and IL-6 [134,135]. Pro-inflammatory cytokines cross the blood–brain barrier and reach the brain, causing NF-κB activation in microglial cells in the brain, leading to neuroinflammation [133,135]. The inflammatory response in adipose tissue is related to hippocampal plasticity and cognitive function impairment caused by obesity.

### 4.5. Oxidative Stress and Mitochondrial Dysfunction

Oxidative stress is an imbalance between the oxidation molecules produced by cells and the antioxidant system that neutralizes them. Oxidative stress and inflammation are mutually reinforcing harmful reactions. Pro-inflammatory cytokines secreted by fat activate signaling pathways that can stimulate the production of reactive oxygen species (ROS) by enzymes such as NADPH oxidase, generating superoxide radicals and hydrogen peroxide [136]. A high-fat diet increases the production of acetyl-CoA and NADH, promoting an increase in the electron transport chain in mitochondria and subsequent production of ROS, leading to oxidative stress [137]. ROS, especially hydroxyl radicals, can oxidize proteins, damage membrane lipids and DNA, increasing the risk of cognitive dysfunction diseases [137]. In the central nervous system, nitric oxide synthase (NOS) is also activated, producing nitric oxide, which generates peroxynitrite anions, nitration of proteins and protein damage [117]. Increased NOS activity is associated with increased calcium and excitotoxicity. Inflammation is related to mitochondrial dysfunction, which leads to decreased ATP levels and increased ROS generation, thereby enhancing oxidative stress [117,138]. The increase in oxidative status activates transcription factors such as NLRP3 inflammasome and NF-κB, which in turn induce the synthesis of more pro-inflammatory cytokines that activate immune cells, thereby sustaining the damage of oxidative stress and inflammation [117,137]. Moreover, obesity increases the number and accumulation of senescent cells in the brain, inducing neuroinflammation and forming a vicious cycle that exacerbates inflammation and oxidative stress, and is associated with age-related cognitive dysfunction diseases [117]. Correlative data have shown that mice fed a high-fat diet have higher levels of ROS, superoxide and peroxynitrite in the brain, leading to lower levels of brain-derived neurotrophic factors and decreased cognitive ability [135]. High-fat diet-induced impairment of nuclear factor-kappaB signaling and increased oxidative stress also contribute to cognitive decline in the aging brain [139].

Mitochondrial dysfunction is associated with inflammation and oxidative stress, where the generation of reactive oxygen species (ROS) exceeds the physiological antioxidant protective activity. Excessive ROS production can cause mitochondrial DNA damage, lipid peroxidation, and oxidative phosphorylation. Animal and clinical evidence from animal models has shown that obesity can cause mitochondrial dysfunction in the brain. As neurons have high energy demands, mitochondrial activity plays an important role in maintaining sustained energy supply. Impaired mitochondrial activity can lead to neuronal damage and functional impairment, resulting in neurotoxicity [137]. Obesity-related learning and memory impairments are associated with decreased mitochondrial density and ATP formation in neurons [138]. Transcriptional co-activator peroxisome proliferator-activated receptor-gamma coactivator-1 alpha (PGC-1α) is the major regulator of mitochondrial biogenesis. PGC-1α mediates the formation and maintenance of dendritic spines in neurons. Silencing PGC-1α not only reduces dendritic density in primary hippocampal neurons but also reduces mitochondrial density and ATP formation. In contrast, activation of the BDNF/PGC-1α cascade signal can promote synaptic formation in hippocampal neurons [140]. Sirtuins (SIRT) are a cytoplasmic and nuclear NAD+-dependent deacetylase that regulates transcription factors by histone deacetylation [138]. Mitochondrial activity and oxidative phosphorylation in skeletal muscles are regulated by the adiponectin receptor 1/AMP-activated protein kinase/SIRT1/PGC-1α signaling pathway [141]. Evidence from animal models has shown that AdipoR1 knockout disrupts the signal pathways that are critical to mitochondrial function, including oxidative phosphorylation, TCA cycle, and β-oxidation [141]. Adiponectin deficiency induces Aβ oligomerization, reduces mitochondrial membrane potential in hippocampal neurons, and can be restored by activating adiponectin receptor signaling [138]. These findings suggest that neuronal mitochondria participate in regulating cognitive function by modulating synaptic plasticity through the PGC-1α signaling pathway.

This figure illustrates the progressive pathway linking adipose pathology to cognitive impairment. Adipose tissue can secrete lipids, proteins, extracellular vesicles, metabolites and non-coding RNAs to communicate with the central nervous system by influencing BBB permeability, synaptic remodeling, hippocampal neurogenesis. etc. The cumulative effect is hippocampal and cortical dysfunction, manifesting as deficits in learning, memory, and executive function.

## 5. Future Perspectives and Conclusions

This review consolidates evidence for the adipose–CNS axis as a critical integrator of metabolic and neurological health. Understanding the adipose–CNS axis opens novel therapeutic avenues. Strategies can target different nodes of the axis:

(1) Source Control: Reducing adipose inflammation through weight loss (lifestyle, pharmacotherapy, or bariatric surgery) remains foundational. Bariatric surgery, for instance, reverses neurogenic obesity patterns after spinal cord injury.
(2) Signal Modulation: Neutralizing specific deleterious mediators (e.g., anti-IL-1β therapies) or boosting protective ones (e.g., adiponectin sensitizers) are active areas of research. Nutritional interventions, such as omega-3 polyunsaturated fatty acid supplementation, show promise in modulating pain pathways, although high ω-6 intake may exacerbate pain.
(3) Neural Circuit Intervention: Modulating the sympathetic or sensory innervation of fat pharmacologically or via bioelectronic medicine could normalize adipose function.
(4) CNS Protection: Compounds that mitigate neuroinflammation, oxidative stress, or mitochondrial dysfunction, such as GlyNAC supplementation, which improves glutathione deficiency and cognition, may be beneficial.
(5) Regenerative Approaches: Interestingly, autologous fat grafting has shown correlation with improvement in localized pain syndromes, potentially via adipose-derived stem cells, highlighting a paradoxical therapeutic use of adipose tissue itself.

At the same time, we believe that, in order to implement the above treatment strategies, there are still some experimental or planning options that need to be further explored in the future:

(1) Temporal Dynamics: Delineate the sequence of events from early adipose expansion to the establishment of chronic pain and cognitive decline.
(2) Spatial Specificity: Determine if different adipose depots (visceral vs. subcutaneous) release distinct EV cargos or signals with selective effects on specific brain regions.
(3) Mechanistic Resolution: Employ single-cell and spatial transcriptomics in both adipose and CNS tissues to map precise cellular dialogues.
(4) Sex-Specific Therapeutics: Develop and test interventions that account for the sexually dimorphic nature of adipose signaling.
(5) Causal Validation in Humans: Translate mechanistic insights from animal models using human biomarkers, neuroimaging, and targeted clinical trials.

In conclusion, adipose tissue is far more than a passive energy reservoir. It is a dynamic, innervated, and secretory organ that maintains a constant dialogue with the CNS. The dysregulation of this dialogue in obesity creates a pathological feed-forward loop that drives both chronic pain and cognitive impairment. Moving forward, a mechanistic understanding of this axis will be essential for developing effective, integrated strategies to combat the overlapping epidemics of metabolic and neurological disease.

## Figures and Tables

**Table 1:** Key Adipose-Derived Mediators in Pain and Cognitive Dysfunction.

Adipokine	Pain	Cognition
Leptin	Pro-nociceptive: - ↑ Spinal NMDAR → neuropathic pain - Activates macrophages to release MMP-9/iNOS → hyperalgesia - ↑ Serum leptin correlates with chronic pain	Biphasic regulation: - Physiological: Promotes hippocampal neurogenesis & synaptic plasticity (via PI3K/AKT) - Pathological (obesity): Induces neuroinflammation → cognitive impairment
Adiponectin	Analgesic: - Intrathecal injection inhibits inflammatory hyperalgesia - Inhibits TRPV1/p38 MAPK → alleviates neuropathic pain	Neuroprotective: - Activates p38 MAPK/GSK-3β/β-catenin → hippocampal neurogenesis - ↓ Aβ aggregation - Low doses improve cognition; high doses suppress neurogenesis
Resistin	Pro-nociceptive: - Stimulates macrophages to release TNF-α/IL-1β → peripheral hyperalgesia - ↑ Synovial fluid levels correlate with pain severity in OA	Impairs cognition: - Promotes neuroinflammation via microglial NF-κB activation - Exacerbates related brain inflammation
NGF	Pro-nociceptive: - Directly induces peripheral/CNS hyperalgesia - TNF-α ↑ adipocyte NGF→ pain exacerbation	Biphasic regulation: - Modulates synaptic plasticity (indirect evidence) - Impaired BBB transport in obesity → cognitive decline
Inflammatory Cytokines (IL-1β, TNF-α)	Pro-nociceptive: - Sensitize nociceptors, sustain chronic pain	Impair cognition: - IL-1β disrupts hippocampal synaptic plasticity - Excessive TNF-α disrupts synaptic homeostasis

## Data Availability

No new data were created or analyzed in this study.
